# Alterations in Epithelial Cell Polarity During Endometrial Receptivity: A Systematic Review

**DOI:** 10.3389/fendo.2020.596324

**Published:** 2020-10-27

**Authors:** Sarah Whitby, Wei Zhou, Evdokia Dimitriadis

**Affiliations:** ^1^Department of Obstetrics and Gynaecology, University of Melbourne, Parkville, Melbourne, VIC, Australia; ^2^Gynaecology Research Centre, Royal Women’s Hospital, Parkville, Melbourne, VIC, Australia

**Keywords:** plasma membrane transformation, endometrium, epithelial-mesenchymal transition, endometrial luminal epithelium, cell polarity, receptivity, implantation

## Abstract

**Background:**

Abnormal endometrial receptivity is one of the major causes of embryo implantation failure and infertility. The plasma membrane transformation (PMT) describes the collective morphological and molecular alterations occurring to the endometrial luminal epithelium across the mid-secretory phase of the menstrual cycle to facilitate implantation. Dysregulation of this process directly affects endometrial receptivity and implantation. Multiple parallels between these alterations to confer endometrial receptivity in women have been drawn to those seen during the epithelial-mesenchymal transition (EMT) in tumorigenesis. Understanding these similarities and differences will improve our knowledge of implantation biology, and may provide novel therapeutic targets to manage implantation failure.

**Methods:**

A systematic review was performed using the Medline (Ovid), Embase, and Web of Science databases without additional limits. The search terms used were “(plasma membrane* or cell membrane*) and transformation*” and “endometrium or endometrial.” Research studies on the PMT or its regulation in women, discussing either the endometrial epithelium, decidualized stroma, or both, were eligible for inclusion.

**Results:**

A total of 198 articles were identified. Data were extracted from 15 studies that matched the inclusion criteria. Collectively, these included studies confirmed the alterations occurring to the endometrial luminal epithelium during the PMT are similar to those seen during the EMT. Such similarities included alterations to the actin cytoskeleton remodeling of adherens junctions, integrin expression and epithelial-stromal communication. These were also some differences between these processes, such as the regulation of tight junctions and mucins, which need to be further researched.

**Conclusions:**

This review raised the prospect of shared and distinct mechanisms existing in PMT and EMT. Further investigation into similarities between the PMT in the endometrium and the EMT in tumorigenesis may provide new mechanistic insights into PMT and new targets for the management of implantation failure and infertility.

## Introduction

Embryo implantation failure is a major cause of infertility, which affects approximately one in six couples worldwide (1). While assisted reproductive technology has enabled many couples to conceive, only approximately 25% of embryos transferred successfully implant (2, 3). It is well established that a successful implantation is reliant on a receptive endometrium, a functional blastocyst, and synchronized cross-talk between these two (2, 4). High-quality embryos alone do not necessarily lead to a successful implantation which remains a significant bottleneck for *in vitro* fertilization (IVF) treatment. There is a recent focus on examining changes in the endometrium that are required for successful implantation. The endometrium undergoes substantial remodeling throughout the menstrual cycle for the purpose of becoming adequately prepared or receptive to an implanting blastocyst. The most luminal portion of the endometrium, namely the functionalis, undergoes ovarian-steroid-hormone-mediated alterations across the average 28-day menstrual cycle to become receptive. The functionalis is comprised of the glandular epithelium (GE), luminal epithelium (LE) and stroma, and progesterone-mediated structural and functional alterations must occur to become optimally receptive during the “window of implantation” (WOI), which spans day 20–24 of the menstrual cycle. Such alterations include the increased secretory capacity and tortuosity of the GE, the acquisition of adhesive and loss of inhibitory cellular components within the LE, and stromal cell decidualization. Sequential exposure of estrogen and progesterone to the human endometrium is required for attainment of receptivity (5). Both steroid hormones act *via* their respective nuclear receptors to regulate transcription of a large number of genes, the products of which can act to regulate expression of additional genes in a downstream, autocrine, paracrine, or endocrine manner (6).

Although progesterone is the main inducer of these changes, progesterone itself is responsible for the down-regulation of its own receptor in the endometrial epithelium during the WOI (7). Indeed, progesterone receptor (PR) down-regulation is crucial for cell-signaling and cytokine expression in mice (8), and failure of PR down-regulation is associated with luteal phase defects (9) and endometriosis (10) in women. Progesterone-medicated regulation of receptivity within the epithelium during the mid-secretory phase is therefore mostly attributed to progesterone induced paracrine factors produced in the stroma. On the other hand, estrogen receptor (ER) expression is reduced across the secretory phase in all components of the human endometrium (11). As such, it is likely that estrogen may have a negative impact on endometrial receptivity. This inhibitory role is supported by the appearance of integrin αVβ3, critical for adhesion, with estrogen down-regulation during the WOI (2). The main action of estrogen may be to prime the endometrium during the proliferative phase, leading to the induction of its proliferation and the recruitment of PR (5). While it may not play a role during the WOI, estrogen may contribute to the early alterations occurring within the endometrium necessary to confer receptivity. The observation that luteal-phase estrogen antagonism disrupts the secretory development of the endometrium supports this (12).

During endometrial remodeling in humans there is increased secretion by glandular epithelial cells, coinciding with the loss of cell polarity in the endometrial LE. Cell polarity is defined as the asymmetrical distribution of cellular components (13). Polarity is a fundamental feature of almost all cells, and has a differential manifestation depending on the function of each cell type. Cell polarity is established by molecular polarity determinants which localize to each cellular domain and direct the functioning of intracellular structures and systems (14). Epithelial cells exhibit apical-basolateral and planar polarity (13), which direct the formation of a functional barrier and regulate cell orientation, tissue structure, cell-cell adhesion, and cell signaling. This is also true for the endometrial epithelium, in which cell polarity contributes to a repulsive apical surface, regulates paracellular movement, directs cell-cell signaling, and maintains cytoskeletal integrity. However, as the endometrial LE is the first point of contact with the implanting blastocyst, the maintenance of endometrial LE polarity would lead to a mutual repulsion between the endometrium and polarized trophectoderm, and therefore the endometrium would not be receptive. Endometrial LE polarity must be lost during the mid-secretory phase of the menstrual cycle for the endometrium to become receptive and allow implantation to proceed. Loss of cell polarity involves the endometrial LE undergoing both morphological and molecular changes. These apical changes include those of the microvilli, cell-surface markers, cellular junctions, and cytoskeletal molecules (15–17). To facilitate invasion, the LE weakens the lateral epithelial surface interactions by reducing the expression of luminal adherens junction proteins and disturbs focal adhesions to the basal lamina. Collectively, these LE alterations lead to the “plasma membrane transformation” (PMT) (17–19) which is essential for implantation.

**Figure 1:**
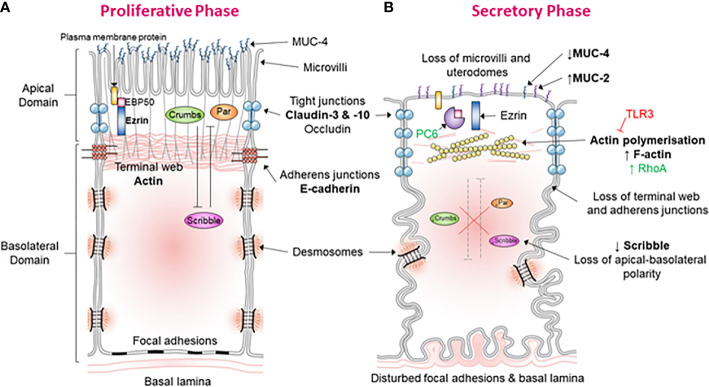
Morphological and molecular alterations during the “plasma membrane transformation” in human endometrium. **(A)** Endometrial epithelium of the proliferative phase. This figure shows the morphology and molecular mechanisms of epithelial cells in the estrogen-dominant proliferative phase. The microvilli are intact, tight junctions (blue) are few and located apically, abundant MUC-4 is present apically, adherens junction are with an associated terminal web, the terminal web is associated with plasma membrane proteins *via* ERM proteins, desmosomes, and focal adhesions are undisturbed, the basal lamina is relatively straight and polarity determinants have established apical-basolateral polarity. **(B)** Endometrial epithelium of the secretory phase. This figure demonstrates the epithelial morphology and molecular mechanisms in the progesterone-dominant secretory phase. The microvilli have been lost, tight junctions (blue) are up-regulated and have moved down the lateral membrane, MUC-4 expression has been down-regulated and MUC-2 up-regulated, actin polymerization of the terminal web has led to terminal web disruption and dissociation from adherens junctions, PC6 has cleaved ERM leading to dissociation of plasma membrane proteins with the terminal web, desmosomes, and focal adhesions are disturbed, the basal lamina has become tortuous, and down-regulation of the polarity determinant Scribble has led to the loss of cell polarization.

In keeping with this notion, it has been validated that dysregulation of PMT in the mid-secretory phase leads to implantation failure and infertility. Dysregulation of apical alterations has been best studied in this case. Apical alterations confer the adhesive capacity of the LE during the mid-secretory phase, and these include modifications of the apical morphology, glycoprotein composition, and cell-surface charge (**Figure 1**) (19). Notable examples for morphological change include pinopods which are large bleb-like protrusions extending beyond the microvilli (2). These are up-regulated during the mid-secretory phase and are thought to contain specific receptors required for blastocyst adhesion at the apex (20). A reduced formation of pinopods has been implicated in at least some clinical cases with unexplained infertility (21). The apical surface of the LE also features a change of proteins that facilitate the adhesive capability. Reduced expression of leukemia inhibitory factor (LIF) receptor is observed in the apical LE of women with unexplained infertility (21) and such reduction impacts LIF dependent pathways involved in regulating endometrial receptivity. LIF is thought to be a key regulator of endometrial receptivity in mice and humans and therefore vital for implantation. LIF protein expression has been found to be most prominent across the mid- and late-secretory phases in the GE and LE of fertile women (22). LIF messenger RNA (mRNA) is shown to be up-regulated in the LE, GE, and decidua across the secretory phase (23). The lack of adequate up-regulation of LIF secretion in patients with unexplained fertility and recurrent implantation failure supports the crucial role of LIF to implantation (2). Moreover, *in vivo* treatment of the endometrial GE with mifepristone, a progesterone antagonist, reduces LIF expression at the expected time of implantation (24), showing LIF expression may be regulated at least in part by progesterone. Together, these indicate a crucial role for LIF in endometrial receptivity. Indeed, LIF has been shown to interact with various other secreted factors to regulate endometrial receptivity, decidualization, blastocyst activation, embryo-endometrial interaction, endometrial invasion, and immune modulation, all crucial to implantation (25).

Reductions in cell-surface negativity with concomitant modifications to the glycocalyx layer also improve the adhesiveness of the endometrial LE (2, 16). The major glycocalyx subunit known to be lost during the mid-secretory phase is mucin 1 (MUC-1) (26). MUC-1 is well-known as an anti-adhesive protein that expressed extensively on the apical surface of uterine luminal and GE as well as cervical and vaginal epithelium (27). The extracellular domain of MUC-1 is enriched with proline residue which endow this glycoprotein with rigidity to extend much further compared to other transmembrane proteins (28). Upon embryo implantation in mice, MUC-1 expression is specifically reduced in the LE and in humans is down-regulated in primary human endometrial epithelial cells at the site of human blastocyst attachment *in vitro* attachment (27, 29). Investigation of clinical samples reveals that abnormal endometrial MUC-1 levels are likely regulated by progesterone and are associated with implantation failure (30).

PMT includes various modifications to the lateral and basal plasma membrane during the mid-secretary phase to facilitate implantation (**Figure 1**). Several lateral junction types have been revealed with changes upon entering the mid-secretory phase. A progesterone-mediated increase in junctional strand cross-linking occurs within tight junctions, producing morphologically “tighter” junctions (15, 17, 19). Tight junctions also increase in depth by three-fold down the lateral membrane, and this, along with the increase in junctional tightness, is thought to decrease paracellular movement of luminal fluid which is essential to blastocyst development (15, 17, 19). Immediately below are adherens junctions, which are associated with an actin-rich terminal web and are displaced with the downward movement of tight junctions (17). A progesterone-mediated transient increase in intracellular calcium is thought to suppress E-cadherin expression, the main constituent of adherens junctions. Similarly, desmosomal protein expression is reduced during the window of implantation (19). Desmosomes are intercellular junctions providing strong adhesion between adjacent cells by linking the intermediate filaments *via* cadherin-rich protein plaques in the plasma membrane. Collectively, these alterations in cellular junctions across the lateral plasma membrane facilitate blastocyst development, apical flattening, and cell individualization.

Similarly alterations to the basal plasma membrane also occur. A progesterone-mediated decrease in focal adhesions occurs and serves to anchor the endometrial LE to the extracellular matrix and basal lamina. Integrins have been implicated in this process to facilitate the penetration of the trophoblast through the LE into the stroma (19). Although dysregulation of lateral and basal plasma membrane modifications is associated with clinical infertile cases (31), there is limited evidence of the precise mechanisms involved in these events and the overall PMT process.

Resolving these questions has potential benefits of informing novel approaches for fertility intervention. However, current understanding of PMT in regulating endometrial receptivity remains incomplete. “Epithelial-mesenchymal transition” (EMT) is a similar process to PMT and describes the loss of apical-basolateral polarity in epithelial cells and the acquisition of a mesenchymal phenotype, typically associated with tumorigenesis. Specifically, EMT involves the disruption of cell-cell junctions, an acquisition of front-rear polarity, cytoskeletal and morphological alterations, enhanced motility, the down-regulation of epithelial and up-regulation of mesenchymal gene expression, and enhanced extracellular matrix degradation (32). These changes result from transcription program switching induced by various interconnected transduction pathways and signaling molecules. Some examples of these include transforming growth factor-β, bone morphogenetic protein, Wnt-β-catenin, Notch, Hedgehog, steroid receptor coactivator, small GTPases, integrins, beta-catenin, Src family kinases, and receptor tyrosine kinases (33, 34). These factors alter gene expression to facilitate increased migration, invasiveness, and resistance to apoptosis in tumor cells, thereby enhancing invasion into local tissue and metastasis (35). Multiple parallels have been drawn between PMT and EMT, specifically with the loss cell polarity which raises the prospect of shared mechanisms between these two processes. The dysregulation of tight junctions and the destabilization of adherens junctions are some examples (19, 32). Since EMT has been extensively studied in humans, and the events of the EMT and its regulation are well characterized, a systematic comparison between PMT and EMT may improve knowledge of endometrial receptivity in women, and therefore provide potential novel biomarkers for infertility or therapeutic targets to manage implantation failure in IVF.

### Research Question

To the best of our knowledge, there have been no systematic reviews to date summarizing the events of the PMT in the human endometrium. Therefore, the aim of the present review was to evaluate the events of the PMT in women and compare these results with the key events described in the EMT.

## Methods

### Data Sources and Search Strategy

The Medline (Ovid), Embase, and Web of Science databases were searched for articles published on or before the 27^th^ of May 2020. The search terms used were “(plasma membrane* or cell membrane*) and transformation*” and “endometrium or endometrial.” No additional limits were used.

### Inclusion and Exclusion Criteria

Human studies assessing the PMT or its regulation in women, discussing either the endometrial epithelium, decidualized stroma, or both, were eligible for inclusion. Decidualized stroma was included as it can act on the LE *via* direct or indirect effects and the function of decidualized stroma on PMT is just beginning to be realized. Inclusion criteria included: 1) articles discussing the PMT in the context of the endometrial epithelium, decidualized stroma, or both; 2) experimental designs; 3) studies including women as subjects. Exclusion criteria included: 1) an animal other than human without “human” in the title; 2) articles discussing gynecological cancer, alterations to the trophoblast, or early pregnancy changes without discussing the PMT; 3) conference abstracts, reviews or lack of available full text; 4) use of exclusively animal subjects. Sample size is indicated in **Tables 1** and **2**.

**Table 1:** Summary of human endometrial epithelial cell studies included.

**Author, year**	**Sample type and size**	**Compare with infertile sample**	**Cycle stage**	**Relevant results**
Heneweer et al., 2002 (36)	Cell line: n = 6-15/group	n/a	RL95-2 cell line (MS)	F-actin increases in the apex of cells upon spheroid adhesion, this actin remodeling most likely regulated by RhoA
Montazeri et al., 2015 (37)	Cell line: n = 3	n/a	RL95-2 cell line (MS)	TLR-3 activation led to reduced spheroid adhesion, actin polymerization and CD98 and β3-integrin expression
Martin et al., 2000 (38)	Primary cells: unknown; Cell lines: unknown	no	Endometrial tissue (all L); RL95-2 cell line (MS); Hec-1A cell line (P)	RL95-2 cells have an adhesion rate of 81% and are associated with reduced ezrin and absent moesin expression compared to HEC-1A cells (46%). Primary cells have an intermediate adhesion rate (67%)
Heng et al., 2011 (39)	Tissue: n ≥ 48; Cell line: unknown	Yes	Endometrial tissue (P n => 10, MS n = 25); Hec-1A cell line (P)	Reduced PC6, which cleaves the scaffolding protein EBP50, affects the interaction of EBP50 with ezrin, EBP50/ezrin cellular localization and cytoskeleton-membrane connections
Demir et al., 2002 (40)	Tissue: n = 18	No	Endometrial tissue (ES n = 4); Decidua (ED n = 6, LD n = 8)	Morphological and molecular changes in the LE and GE play a role in cellular defense and limiting trophoblastic invasion during early pregnancy compared to ES endometrium
Bentin-Ley et al., 2000 (41)	Primary cells: unknown	No	Endometrial tissue (LH+5 to LH+7)	Blastocysts adhere and invade the endometrium *via* intrusive penetration simultaneously, leading to syncytium formation
Kabir-Salmani et al., 2005 (42)	Tissue: n = 23	No	Endometrial tissue (EL n = 5, ML n = 10, LL n = 8)	LIF expression increased in uterodomes during the MS phase, and was co-localized with markers of exocytosis
Alameda et al., 2007 (43)	Normal and pathological tissue: n = 98 (n = 79 pathological)	No	Endometrial tissue: Normal (P n = 11, S n = 8); 44 endometrial hyperplasia and 35 endometrial adenocarcinomas	MUC-4 is detected in the P GE (36.3%) but is down-regulated in the S (12.5%) wherein MUC-2 is up-regulated (37.5%)
Whitby et al., 2018 (44)	Tissue: n ≥ 10/group; Primary cells (SCs): n = 5; Cell line: n = 4	no	Endometrial tissue (P n = 10, ES n = 10, MS n = 10, LS n = 10); SCs (P or ES n = 5); ECC-1 cell line (LE)	TER (as a measure for polarity) was reduced in ECC-1 cells treated with E2 and P4. Stardust, atypical PKC, Crumbs and Scribble are reduced in the LE and Scribble increased in SCs in S *versus* P endometrium. Scribble KD in ECC-1 cells and SCs enhanced spheroid adhesion and down-regulated decidualization, respectively
Greening et al., 2016 (45)	Tissue: n = 5/group; Primary cells: n = 6; Cell line: n = 3	no	Endometrial tissue (P, S, and T1); EECs: unknown; ECC-1 cell line (LE)	157 cellular proteins are altered with progesterone, and 193 are further altered with hCG. 123 proteins are altered in the secretome with progesterone, and 43 are further altered by hCG
Van Sinderen et al., 2017 (46)	Tissue: n ≥ 4/group; Flushings: n = 14; Cell line: n = 3/group	yes	Endometrial tissue (ES or MS); Uterine lavage (S); RL-95 cell line (MS)	Infertility is associated with increased soluble DLL1, which reduces epithelial adhesive capacity *via* HES1 mRNA down-regulation, and increased ADAM17 expression in the LE, which cleaves DLL1 to form its soluble form. Soluble DLL1 may inhibit Notch signaling

n/a, not applicable; L, luteal phase; P, proliferative; S, secretory; T1, first trimester; LE, luminal epithelium; GE, glandular epithelium; ES, early-secretory; MS, mid-secretory; LS, late-secretory; EL, early luteal; ML, mid-luteal; LL, late-luteal; ED, early decidua; LD, late decidua; EECs, endometrial epithelial cells; SCs, stromal cells; LH, luteinizing hormone; TLR-3, toll-like receptor-3; CD98, 4F2 cell-surface antigen heavy chain; RhoA, Ras homolog family member A; hCG, human chorionic gonadotrophin; MUC, mucin; PC6, proprotein convertase 5/6; EBP50, ezrin-radixin-moesin binding phosphoprotein 50; LIF, leukemia inhibitory factor; DLL1, delta-like ligand 1; HES1, hairy and enhancer of split-1; ADAM17, “a disintegrin and metalloprotease” protease-17; TER, trans-epithelial resistance; IHC, immunohistochemistry; E2, estrogen; P4, progesterone; atypical PKC, atypical protein kinase C; KD, knock-down. Cycle stage for cell lines is representative.

**Table 2:** Summary of human endometrial stromal cell studies included.

Author, year	Sample size (fertility)	Cycle stage	Relevant results
Gililland et al., 1992 (47)	n = 7 (fertile)	Endometrial tissue (P n = 2; ES n = 3, LS n = 2)	Specific high affinity ANP-R were identified in endometrial tissue, and may regulate cell function or development *via* cGMP
Schumann et al., 2015 (48)	Decidua, n = 4; Endometrial tissue: n = 6 (fertile)	Decidua (7–9 weeks pregnant); Endometrial tissue (P n = 3, S n = 3)	Claudin-3 and -10 were identified in the endometrial epithelium but not early pregnancy decidua, and claudin-3 in extravillous trophoblast
Murakami et al., 2014 (49)	n = 43 (unknown)	Endometrial tissue (6–10 days post-LH surge)	SUSD2 expression alters with cell-cell contact and Notch signalling, and thus alters the perivascular secretome upon decidualization
Szwarc et al., 2018 (50)	Unknown (unknown)	Endometrial tissue (all P)	A subset of stromal cell genes regulated by PGR during decidualization also requires SRC-2, for example retinoid signaling

P, proliferative; ES, early-secretory; LS, late-secretory; S, secretory; LH, luteinizing hormone; ANP-R, atrial naturetic peptide-receptors; cGMP, cyclic guanosine monophosphate; SUSD2, sushi domain containing 2; PGR, progesterone receptor; SRC-2, steroid receptor coactivator-2.

### Data Extraction and Study Selection

Data were then extracted from the identified articles. Quality assessment was performed independently by two reviewers (SW and WZ). If there was disagreement, a third reviewer (ED) was consulted. The analysis followed the PRISMA statement for systematic reviews.

## Results

**Figure 2:**
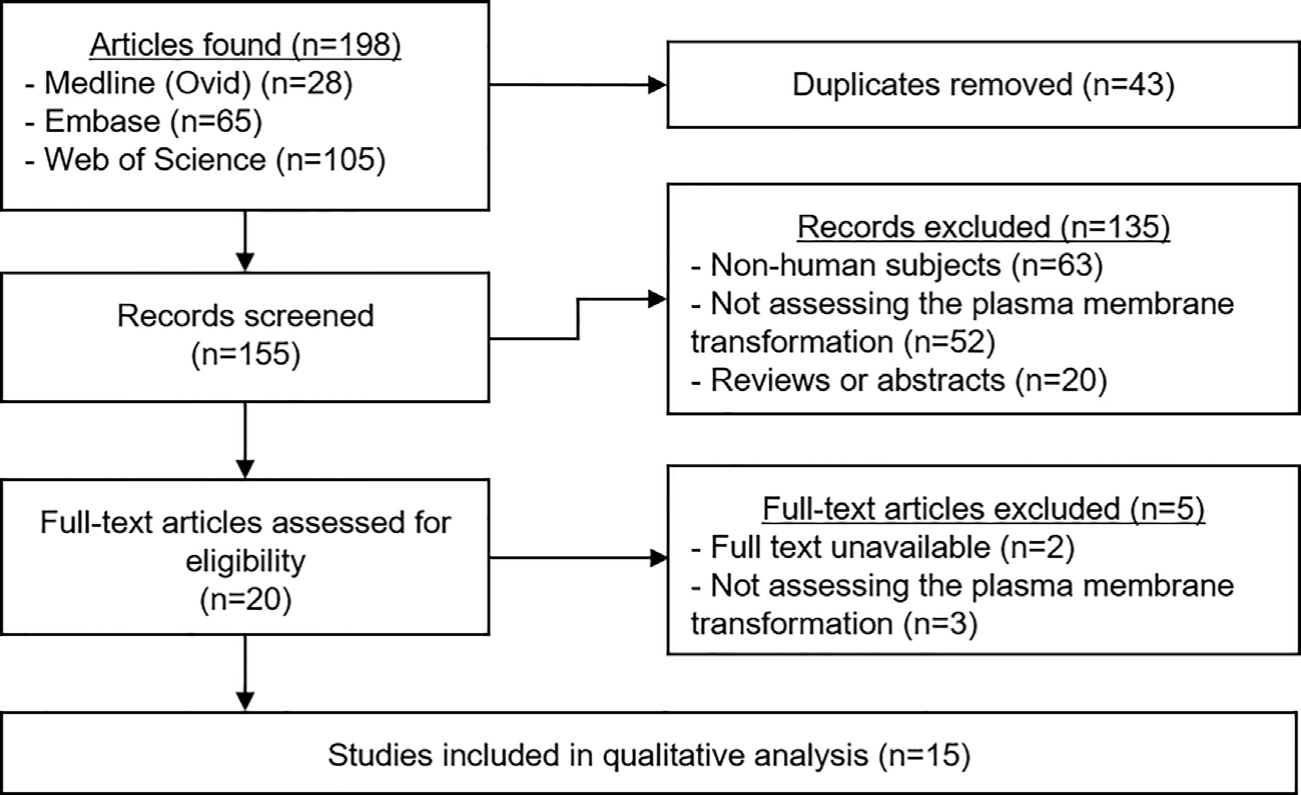
PRIMSA Flowchart. The above diagram shows the different phases of the systematic review process for this article as per the PRISMA guidelines.

A total of 198 articles were identified (**Figure 2**). Duplicates were removed, leaving 155 manuscripts for screening. Titles and abstracts were read carefully, and application of the selection criteria led to the exclusion of 135 articles. The full text of the remaining 20 papers was read to ensure their relevance, and a further five studies were excluded. The data from the remaining 15 studies were extracted and the quality of each was analyzed.

The results of the studies on the events of the PMT in human endometrial epithelial cells and endometrial stromal cells are summarized in **Tables 1** and **2**, respectively. Alameda et al. (43) identified the expression of MUC-2 was increased in secretory *versus* proliferative phase glandular endometrial epithelial cells. Conversely, MUC-4 was reduced in the GE (43). The cell-surface marker atrial natriuretic peptide (ANP) receptor was identified within secretory phase fertile endometrial tissue compared to proliferative phase tissue (47). Schumann et al. (48) revealed claudin-3 and claudin-10 were expressed in both the LE and GE across the proliferative and secretory phases, however no significant differences were seen across the cycle. Trans-epithelial resistance (TER), a sensitive indicator of cell-cell barrier function and therefore a proxy measure of polarity, was also found to be reduced in the endometrial epithelial cell line, ECC-1, cells treated with combined estrogen and progesterone, which mimics mid-secretory phase endometrial LE (44). Moreover, Whitby et al. (44) revealed the expression of the polarity determinants known to direct apical-basolateral polarity in tumorigenesis, namely Stardust, atypical protein kinase C (PKC), Crumbs and Scribble, were increased in ECC-1 cells across the mid-secretory phase. Heneweer et al. (36) found apical F-actin expression and actin polymerization increased in RL95-2 cells (derived from endometrial adenosquamous carcinoma), which models the non-polar endometrial epithelium, upon spheroid adhesion. This may be positively regulated by the Ras homolog family member A (RhoA) GTPase (36), and negatively regulated by Toll-like receptor-3 (TLR-3) (37). Martin et al. (38) revealed RL95-2 cells were also associated with reduced ezrin and absent moesin expression, which otherwise contribute to cytoskeletal stabilization in polarized cells by connecting membrane-associated proteins to actin filaments. Correspondingly, Heng et al. (39) found proprotein convertase 5/6 (PC6) knockdown in HEC-1A cells (endometrial adenocarcinoma cell line), which model the polarized endometrial epithelium, was associated with reduced mouse blastocyst attachment. PC6 cleaves the ezrin-radixin-moesin binding phosphoprotein 50 (EBP50) scaffolding protein and usually facilitates cytoskeletal re-organization during the mid-secretory phase. Montazeri et al. (37) also showed TLR-3 activation, which represents viral-induced immune activation causing a reduced rate of implantation, was also associated with reduced CD98 and β3-integrin expression.

Various other morphological and molecular alterations involved in trophoblastic invasion were characterized within the human endometrial epithelial studies (**Table 1**) (40). Kabir-Salmani et al. (42) found LIF was co-localized with markers of exocytosis in the pinopods of endometrial tissue from fertile women using immunohistochemistry. Greening et al. (45) found a significant alteration in the intracellular protein profile and secretome of ECC-1 cells after supplementation with estrogen and progesterone compared to estrogen alone. Specifically, Van Sinderen et al. (46) showed soluble delta-like ligand 1 (DLL1) was increased in the uterine lavage of infertile patients, which was associated with increased intracellular “a disintegrin and metalloprotease” protease-17 (ADAM17) expression in endometrial tissue and reduced epithelial adhesiveness of RL95 cells. ADAM17 cleaves DLL1 into its soluble form, which may inhibit Notch signaling and therefore interfere with endometrial receptivity (46). Furthermore, Murakami et al. (49) revealed the expression of sushi domain containing 2 (SUSD2) was found to be altered in mid-luteal phase stromal cells collected from women. This alteration was shown to be associated with cell-cell contact and Notch signaling and facilitated a change in the perivascular secretome. Szwarc et al. (50) identified a subset of stromal cell genes regulated by the progesterone receptor also required the co-receptor Steroid receptor coactivator-2 (SRC-2), such as retinoid signaling.

## Discussion

The loss of endometrial LE polarity during the PMT is a crucial step to attain endometrial receptivity to the blastocyst prior to implantation. This overcomes the mutual repulsion that exists between the otherwise polarized endometrial epithelium and embryonic trophectoderm to facilitate blastocyst implantation. While the events surrounding the loss of LE polarity have not yet been fully characterized, multiple parallels have been drawn between this process and the EMT in tumorigenesis. The present review summarized and analyzed relevant studies within the endometrium to compare these alterations to those seen during an EMT. Collectively, these studies confirmed the morphological and molecular alterations occurring during the PMT (**Figure 1**) are similar to those which occur during an EMT. Such similarities included alterations to the cytoskeleton, remodeling of cell-cell junctions, and cell-surface marker expression (**Table 3**). The endometrial epithelium can be divided into the LE and GE. However, it should be noted that, although there are similarities between the loss of cell polarity within these components of the endometrium across the mid-secretory phase, there are also differences. For example, the localization of some cell-cell junctions along the basolateral membrane and the expression of individual junctional proteins differs between the LE and GE (41). Therefore, while investigating the alterations to the GE across the cycle is important, this review primarily focuses on the LE as the LE is the first point of contact with the implanting blastocyst.

**Table 3:** Summary of alterations during the plasma membrane transformation *versus* epithelial-mesenchymal transition and comparison to animal models.

Category	Alteration	Plasma membrane transformation	Epithelial-mesenchymal transition	Animal implantation models
Apical membrane	Microvilli	Loss of microvilli leads to apical flattening and thus facilitates adhesion	Loss of microvilli causes apical flattening	Loss of microvilli leads to a smooth apical surface in mice (51)
	Actin-rich apical protrusions	Up-regulation of pinopods co-localized with vesicles containing LIF contribute to blastocyst adhesion and implantation	Formation of actin-rich invadopodia co-localized with vesicles containing MMPs aid tumor cell invasion (52)	LIF null mice fail to develop apical pinopods and no implantation is observed (53)
	Integrins	Integrin αVβ3 up-regulation and apical recruitment of integrins contribute to blastocyst adhesion	Increased apical integrin αVβ3 expression and integrin clustering at the leading edge contribute to tumor cell invasion (54)	Increased expression of integrin αVβ3 is observed in mouse luminal surface at the time of implantation and blockade of integrin αVβ3 impairs implantation (55, 56),
	Mucins	MUC-1 and MUC-4 down-regulation and MUC-2 up-regulation facilitates adhesion	MUC-1 and MUC-4 play a crucial role in tumorigenesis, invasion, and metastasis (57)	Substantial reduction of MUC-1 and loss of MUC-4 occur before implantation in rat facilitates adhesion (58, 59),
Lateral membrane	Tight junctions	Tight junctions become morphologically “tighter” and increase in depth 3-fold down the lateral membrane. Claudin-3 and claudin-10 expression is increased	Tight junctions are lost, contributing to cell individualization. Claudin-1, claudin-4 and claudin-10 are differentially expressed (60, 61),	Lateral tight junctions also increase in complexity on the day of implantation in both mouse and rat models. Claudin-3 shift localization to apical surface while Claudin-10 is undetectable in luminal epithelium in mice (48)
	Adherens junctions	Adherens junctions are displaced and E-cadherin down-regulated, contributing to reduced cell-cell adhesion. This is associated with reduced expression of Crumbs, Stardust, αPKC, and Scribble	E-cadherin endocytosis leads to adherens junction dissolution, thus facilitating cell individualization. This may be associated with Crumbs internalization (62)	E-cadherin expression is down-regulated and redistributed from basal and lateral regions to a more apicolateral region (63)
	Terminal web	Actin polymerization, mediated by RhoA, and dissociation with ERM proteins, regulated by PC6, contributes to cytoskeletal alterations and apical flattening	Actin polymerization, mediated by Rho GTPases, and dissociation with ERM proteins may contribute to cytoskeletal alterations and apical flattening (64). However, increased moesin expression may contradict this (65)	Dysregulation of RhoA impair embryo implantation in mice. Inhibit PC6 block embryo implantation in mice (66, 67),
Basal membrane	Focal adhesions	Focal adhesions disruption facilitates cell individualization	Focal adhesions disassembly facilitates cell individualization from invasion (32)	In rat model, focal adhesion proteins disassemble along the basal membrane at the time of implantation (68)
Epithelial-stromal communication	Direct stromal-epithelial signaling	Direct stromal-epithelial cell signaling in the endometrium may regulate the events of the plasma membrane transformation	Direct tumor-stromal communication is involved in the differential regulation of key regulators for tumorigenesis (69)	WNT pathway in the stroma modulates E-cadherin-β-catenin complex in the uterine epithelium, thus regulating apical-basal polarity (70)
	Stromal cell-derived factors	Decidualized stromal cell-derived factors may be involved in the regulation of the plasma membrane transformation	Stromal-cell derived factors contribute to tumorigenesis (71)	Different to humans, the transformation of stroma into secretory decidual cells is triggered by embryo attachment (72)

Similarity and difference compared to PMT were accordingly indicated in green and red in both EMT and animal models. Black indicates incomparable. Abbreviations: LIF, leukemia inhibitory factor; MMPs, matrix metalloproteinases; MUC, mucin; αPKC, atypical protein kinase C; RhoA, Ras homolog family member A; ERM, ezrin, radixin, and moesin; PC6, proprotein convertase 5/6.

### *In vitro* Assays Used to Investigate Plasma Membrane Transformation and *In vivo* Animal Models

The functional studies included in this review are all based on *in vitro* human models. Spheroid adhesion assays are commonly used to test endometrial epithelial cell adhesive capacity. This assay generally involves both endometrial epithelial cells and trophoblast cell spheroids to mimic the blastocyst attachment. The assay uses mechanical force to determine the adhesive capacity of endometrial epithelial cells (73). In some studies, spheroids are replaced with mouse embryos and in very rare cases human IVF blastocysts are co-cultured with a primary human endometrial epithelial/stromal cell bilayer (74). To identify the role of specific molecules in endometrial cell adhesive capacity, the levels of the relevant factor is altered experimentally and their effect on adhesion determined. The xCELLigence technology which incorporates real-time monitoring of cell adhesion is also used as an approach to determine endometrial cell adhesive capacity (46). In this assay, endometrial epithelial cells adhesion is measured either directly on attachment to plastic plates or the plates are coated with various extracellular matrix (ECM) proteins and endometrial epithelial cell adhesion measured *via* electrical impedance in real-time (75). The TER assay is used to measure the integrity of inter-epithelial cell-tight junctions *in vitro* (44). Although these *in vitro* assays provide new mechanistic insight into the PMT process, we caution a direct extrapolation of these models to the equivalent events occurring *in vivo* in the human endometrium. Clinical trials are required to confirm the *in vitro* data in women. Confirmation in animal models especially pre-clinical mouse models serve as a complementary approach. Recent studies in mice have discovered that the structural characteristics including endometrial LE cell polarity of uterine LE have important roles in embryo implantation. Although raised as an exclusion, we have summarized animal implantation models to compare the main results of PMT (**Table 3**) (32, 48, 51–72, 76) and have accordingly added the information into the discussion where relevant.

### The Mucin Family Members

The regulation of mucins on the apical surface of the endometrial epithelium may be different between the EMT and PMT. Alameda et al. (43) found MUC-4 was down-regulated and MUC-2 was up-regulated in secretory *versus* proliferative phase endometrium. Both MUC2 and MUC-4 have been shown to be crucial to successful implantation, polymorphisms of each being linked to endometriosis and infertility (77, 78). MUC-4 has also been implicated in the implantation process in animals, which has been shown to be down-regulated in the endometrium of rodents during the time of implantation (79). As MUC-4 is differentially altered across the cycle, it then leads that ovarian-hormones may play a role in its regulation. This, however, may be an indirect role, as steroid receptors have been shown not to be co-expressed with MUC-4 in the luminal endometrial epithelium of fertile women (80). Furthermore, while the present review did not include studies on MUC-1, this mucin is an anti-adhesive mucin known to be involved in the PMT, as it repels the blastocyst by steric hindrance and must be lost during the mid-secretory phase to facilitate implantation (2). Unlike in animal models, in humans MUC-1 is down-regulated locally in the LE at the site of blastocyst attachment (29) while it is produced in moderate levels in the LE. This suggests that while MUC-1 may be expressed in the endometrial LE during receptivity, communication between blastocysts and LE is critical to down-regulate MUC-1 to enable implantation. Contrary to this knowledge surrounding mucins in the PMT, MUC-4, and MUC-1 have been shown to play a crucial role in the EMT, where these contribute to the malignant transformation, invasiveness and metastatic potential of cancer cells (57). Therefore, these mucins may be differentially regulated in the PMT compared to the EMT. However, Alameda et al. used GE from women with an unknown fertility status. As such, further research is required to fully elucidate the regulation of these mucins in normal endometrial LE during the PMT compared to the EMT.

### Regulation of Tight Junctions Between Plasma Membrane Transformation and Epithelial-Mesenchymal Transition

Of note, the regulation of tight junctions in the PMT compared to the EMT is rather different. While tight junctions increase in depth and tightness during the PMT, they are lost in the EMT (**Table 3**). In polarized epithelial cells, tight junctions prevent the paracellular movement of transported solutes and water (15, 17). Similar changes of tight junctions have also been recorded in both mouse and rat models on the day of implantation (48, 81). Crumbs and Par complexes localize to tight junctions at the apical-basolateral interface to direct the formation of this junctional seal in mammalian cells (82). However, as epithelial cells acquire a mesenchymal phenotype for migration, tight junction dissolution occurs with a concomitant decrease in claudin and occludin expression, which are the main constituents of these cell-cell junctions (32). The difference between these processes is supported by the results from *Schumann* et al. (48), which revealed claudin-3 and claudin-10 expression was maintained in both the LE and GE across the cycle, expression of these being similar in the proliferative *versus* secretory phases. However, claudin-1, claudin-4, and claudin-10 have been shown to be differentially expressed during the EMT in oral lichen planus (60) and ovarian carcinomas (61). Moreover, *Whitby* et al. (44) found constituents of the Crumbs and Par complexes, known to localize to and maintain tight junctions, are down-regulated during the mid-secretory phase. Therefore, it could be that the regulation of tight junctions is fundamentally different in the PMT when compared to the EMT. Given the differential regulation of the Crumbs and Par complexes, this could suggest that claudin expression does not alter in the endometrium across the cycle, but rather a progesterone-mediated alteration in polarity determinant expression of those associated with tight junctions allows for the cellular redistribution of claudins to enable these cellular junctions to increase in number and depth down the basolateral membrane. Indeed, it may even change between species as claudin-10 is undetectable in the mouse uterine LE before implantation (48). However, this discrepancy needs to be further investigated to understand the specific alterations occurring to tight junctions in the PMT compared to the EMT.

The finding by Whitby et al. (44) that TER was reduced with combined estrogen and progesterone treatment following estrogen priming also provides insight into the integrity of tight junctions across the PMT. As stated above, TER is a sensitive indicator of cell-cell barrier function, but specifically TER is a measure of tight junction formation. Therefore, this finding by Whitby et al. may indicate that the mid-secretory phase of the menstrual cycle is associated with a progesterone-mediated reduction in tight junction formation. However, this is at odds with the hypothesis from the previous paragraph and the observation that tight junctions increase in tightness and depth down the basolateral plasma membrane during the PMT. The use of ECC-1 cancer cell line could account for this discrepancy, as tight junctions are known to be lost across the EMT. Indeed, TER has been shown to be reduced in Raf-1 transfected mouse hepatic cell lines (83), with a concomitant decrease in tight junctional proteins. Raf-1 is a signaling pathway known to induce EMT, characterized by the down-regulation of adherens and tight junctions and the re-organization of actin (83). Alternatively, these discrepancies could be explained by the experimental model used. It could be hypothesized that while tight junction formation increases during the mid-secretory phase, locally acting factors derived from the implanting blastocyst may mediate the disruption of tight junctions at the site of implantation to facilitate trophoblastic invasion. Therefore, the model used by Whitby et al. may not have adequately replicated the uterine microenvironment, and therefore did not allow the ECC-1 cells to establish adequate tight junctional formation prior to hormonal treatment. Regardless, this discrepancy requires further research to fully elucidate the function and regulation of tight junctions and the role they play in barrier function during the PMT.

### Comparison of Cytoskeleton Regulation Between Plasma Membrane Transformation and Epithelial-Mesenchymal Transition

Alterations to the endometrial LE cytoskeleton are analogous to those which occur in tumor cells in preparation for invasion and metastasis. The actin network is a dynamic cellular structure which undergoes continuous polymerization and disassembly. Globular-actin monomers, called G-actin, increase in concentration at the cell surface, termed the leading edge, during the EMT and polymerize into filamentous-actin, called F-actin (84). This redistribution of actin disrupts the cortical actin concentration and its regulatory proteins, shifting them to the leading edge of the cancer cells to confer their migratory capacity. As actin polymerization and increased expression of apical F-actin is also observed in the PMT, as shown by Henweer et al. (36), it is likely these events are related. Notably, the redistribution of actin in tumor cells also contributes to the formation of lamellipodia, filopodia, and invadopodia on the leading edge, which are all actin-rich cytoskeletal projections involved in migration and invasion (64, 84). This is similar to the up-regulation of pinopods on the endometrial epithelial surface during the mid-secretory phase, which are also actin-rich projections extending from the apical surface. Moreover, vesicles containing membrane type I-matrix metalloproteinase have been found to be co-localized with exocytotic machinery and key regulators of cell polarity in invadopodia in cancer cells (52), which is similar to the observation by Kabir-Salmani et al. (42). Therefore, both pinopods and invadopodia may play a role in implantation and metastasis, respectively, through the exocytosis of locally acting molecules or factors. Additionally, as RhoA was shown by Heneweer et al. (36) to regulate actin polymerization and F-actin, actin polymerization is also known to be regulated by small Rho GTPases, such as RhoA and Ras-related C3 botulinum toxin substrate 1 (Rac1), in cancer cells. RhoA activates formin proteins at the leading edge to drive actin polymerization near the plasma membrane, and Rac1 regulates the nucleation of actin filaments in lamellipodia *via* the Actin-related protein (ARP) 2/3 complex (85). RhoA and Rac1 are involved in regulating mouse embryo implantation (66, 86). The conditional deletion of Rac1 in the mouse uterine epithelium affects the LE junctional remodeling and prematurely decreases epithelial apical-basal polarity, which leads to defective uterine receptivity and implantation failure. Further analysis on the mechanisms reveals that Rac1 deletion abolishes the activation of Ezrin-radixin-moesin proteins and a similar mechanism has been confirmed in the human Ishikawa cell adhesion *in vitro* (86).

Ezrin-radixin-moesin proteins play a crucial role in the cytoskeletal alterations that occur in the PMT and EMT. These proteins stabilize the cytoskeleton in polarized cells by connecting membrane-associated proteins to cortical actin filaments. Therefore, disruption of these with the loss of cell polarity causes cytoskeletal relaxation and also alters signal transduction. In the PMT, the convertase PC6 cleaves EBP50, which causes the dissociation of EBP50 from ezrin and subsequent destabilization of these from the actin cytoskeleton. Actin is released from the cytoplasmic face of the epithelial cell membrane, which leads to terminal web disruption, adherens junction destabilization and the loss of microvilli, resulting in cell individualization and apical flattening. The temporal and spatial localization of PC6 and EBP50 have been well characterized (67, 87) in mice. Compared to apical membrane staining of EBP50 in the LE during implantation, PC6 expression instead, is restricted to the stromal cells near the embryo attachment site, a location seemingly incompatible to cleave EBP50. However, PC6 inhibition blocks embryo implantation in mice (87). The involvement of EBP50 in human PMT process may be analogous to that in tumorigenesis, as EBP50 depletion in biliary cancer cells has been shown to induce cellular features associated with an EMT (62). These cells show reduced E-cadherin expression and increased activity of the E-cadherin transcriptional repressor, as well as a loss of cell polarity (62). Therefore, EBP50 disruption of biliary cancer cells also contributes to adherens junction dissolution and cytoskeletal re-organization as in the PMT. However, a recent study revealed increased moesin expression regulated adhesion receptor expression and contractile cytoskeletal elements during the EMT in mammalian epithelial cells (65). This difference, however, will need to be further validated. Despite this, there are some interesting parallels between the role of ERM proteins in the rearrangement of the cytoskeleton during the PMT and EMT which warrant further investigation.

Actin polymerization also controls the turnover of cadherin by regulating its endocytosis and therefore regulates adherens junction remodeling during the EMT. The ARP2/3 complex regulating actin polymerization is also thought to play a role in membrane invagination and vesicle formation during endocytosis (88). ARP2/3 is a downstream target of Cell division control protein 42 homolog (Cdc42), which when activated leads to the nucleation of actin at sites of internalization adjacent to adherens junctions, and the subsequent endocytosis of E-cadherin (88). Notably, the disruption of this apical polarity determinant, namely Cdc42, during this process may also mediate the internalization of the Crumbs complex, another apical polarity determinant required for adherens junction stability. Therefore, Cdc42 may also have an indirect effect on E-cadherin endocytosis or disruption (88). The interaction of Cdc42 with Crumbs is consistent with the findings from Whitby et al. (44), which revealed Crumbs was reduced in secretory phase LE. This study also identified Stardust, atypical PKC and Scribble to be reduced in treated cell lines mimicking the receptive LE. In polarized epithelial cells, the apical domain is specified by the Crumbs complex, which contains Stardust in *Drosophila* (14, 89), and Par complex, which contains atypical PKC in *Caenorhabditis elegans*, *Drosophila*, and mammals (13, 14, 82, 89), as well as Cdc42 in *Drosophila*. Conversely, the basolateral domain of a cell is specified by the Scribble complex (13), and negative feedback between these two domains establishes mutually antagonistic interactions to maintain cell polarization (14, 89). It is therefore tempting to postulate that the down-regulation of Stardust, atypical PKC and Scribble also contributes to the loss of cell polarity in the endometrium. Additionally, since the alterations to Crumbs, Stardust, atypical PKC, and Scribble were observed in the progesterone-dominant secretory phase, it could be hypothesized that progesterone, either directly or indirectly, plays a role in the regulation of polarity determinants during the PMT. However, these targets have not been well investigated in mouse uterus and further research is required to validate these hypotheses.

Focal adhesions are also intimately linked to the actin cytoskeleton and are involved with extracellular communication and local signal transduction. Cytoskeletal remodeling during the EMT causes focal adhesion disassembly and the subsequent cellular redistribution or altered expression of the main constituents of these junctions, including focal adhesion kinase, integrin molecules, and vinculin (32). Increased expression of integrin αvβ3 contributes to the pro-invasive functions at the leading edge of lung cancer cells (54). Furthermore, it has been shown to promote the clustering of integrins at the leading edge during the EMT (32). Collectively, these findings reflect the phenomenon of focal adhesion disassembly and apical integrin recruitment recorded in the PMT. This is supported by the work of Montazeri et al. (37), who found TLR-3 activation, known to disrupt implantation, leads to reduced β3-integrin expression. The importance of integrin αvβ3 and focal adhesion disruption on implantation has been confirmed in the animal models, as summarized in **Table 3**.

### Stromal-Epithelial Communication

Stromal-epithelial communication, either direct or indirect, is also essential to facilitate implantation. During the mid-secretory phase in humans, stromal cells transform to secretory decidualized endometrial stromal cells. Stromal cell-epithelial cell signaling, as well as proteins from epithelial or stromal cells contributing to the uterine secretome, are likely to be involved in the PMT. Murakami et al. (49) showed SUSD2-expressing perivascular cells establish specific chemokines and cytokine profiles around the uterine vasculature upon decidualization, which is regulated by cell-cell contact and Notch signaling. Van Sinderen et al. (46) revealed increased soluble DLL1 was associated with reduced cell adhesion and may alter Notch cell signaling (46). Therefore, both SUSD2 and DLL1 may play a role in Notch signaling in the endometrium, which, importantly, is also a well-known inducer of transcription program switching in the EMT. Szwarc et al. (50) revealed progesterone-mediated retinoid signaling was co-regulated by SRC-2, a Src family kinase which is also a known inducer of transcription program switching in the EMT, thereby highlighting another important parallel between these two processes. Moreover, Whitby et al. (44) found Scribble expression was increased in secretory phase stromal cells, which is a key polarity determinant in epithelial cells as discussed above. Although unlike humans, decidualization in mice is triggered by attachment of implanting embryos, genomic analysis on decidualized stromal cells has revealed an overall conserved gene network regulated by steroid hormones in both humans and mice (90). As such, animal models may provide direct *in vivo* evidence to inform conserved stromal-epithelial communication pathways in humans. For example, an *in vivo* functional study using a mouse model has confirmed that uterine deletion of Scribble impaired the formation of the primary decidual zone surrounding the implantation site (91) and compromised implantation. Taken together, these results demonstrate that the regulation of stromal cell signaling in the PMT is similar to that in the EMT, which contributes to epithelial-stromal signaling either directly or indirectly. Proteins in the epithelial cell secretome are also likely involved in the regulation of the PMT. Results from Greening et al. (45) revealed a large number of differentially regulated proteins upon comparing the soluble secretome of ECC-1 cells treated with combined estrogen and progesterone, compared to estrogen alone. Proteins involved in the regulation of cellular adhesion and extracellular-matrix organization were enriched with combined treatment estrogen and progesterone treatment, while the expression of various cytoskeletal and microtubule components was significantly down-regulated in response to combined estrogen and progesterone treatment. Importantly, this further supports the role of progesterone in the indirect regulation of the PMT by mediating factors secreted into the uterine microenvironment. Similarly, tumor-stromal communication occurs indirectly *via* epithelial or stromal cell secreted molecules, or directly *via* cell-cell contact, which contribute to the secretome and is an important source of key regulators for tumorigenesis (71). Therefore, progesterone-mediated stromal cell signaling in the endometrium, either directly or indirectly by contributing to the uterine microenvironment, along with secreted proteins from epithelial cells, is likely vital to the PMT as it is in the EMT.

### The Heterogeneity of the Endometrium

Various markers and gene signatures of the EMT and their association with the tumor microenvironment are currently being evaluated for prognosis prediction (92), for example in head and neck squamous cell carcinoma (93) and glioma (94). Although further research is needed to confirm the use of EMT markers or signatures as predictors of cancer prognosis, this is a promising development for the field. Since multiple parallels have been drawn between the PMT and the EMT, the use of EMT markers or signatures within the context of the endometrium could also provide an interesting avenue for identification of biomarkers of infertility. Furthermore, given the complexity of the regulation of the PMT, while also considering patient-to-patient variation, it is unlikely that a single modulator would be able to predict endometrial receptivity. Therefore, the use of an EMT signature to predict endometrial receptivity could not only provide new biomarkers, but could give a more accurate prediction of infertility. However, there are also many differences between the EMT and PMT, and these are yet to be fully characterized. Therefore, understanding the similarities and differences between these processes in a broader context will improve our knowledge of endometrial receptivity in women, and therefore may provide novel biomarkers for infertility or therapeutic targets to manage implantation failure in IVF.

### Limitations

There were some limitations to process of this systematic review. Firstly, while the term “plasma membrane transformation” was introduced by Murphy et al. (95) and has gained wide acceptance, some studies relevant to this review may not have used this terminology. Despite the optimization of the search terms used here, the possibility that some relevant studies have not been included cannot be ignored. Indeed, the lack of studies discussing alterations to desmosomes or intermediate filaments during the PMT supports this. This review also limited the included studies to those in women. While this ensured results were targeted to the unique implantation biology of humans, the PMT is not well characterized in women due to the difficultly of studying implantation biology directly. Although this likely limited the number of studies included in this review, it was necessary in order to identify gaps in the current literature in humans.

Limitations also existed with the data itself. Each included study discussed a different aspect of the PMT, making it difficult to draw well-substantiated conclusions. Moreover, the included manuscripts often used small sample sizes and varying study subjects, including cell lines, primary tissue, and uterine lavage, which also made drawing comparisons difficult. Different endometrial epithelial cell lines have differing phenotypical and molecular characteristics. For example, while the RL95-2 and ECC-1 cell lines are both considered receptive and both express estrogen and progesterone receptors (96, 97), their cellular makeup is likely still different because they a derived from endometrial adenosquamous carcinoma and endometrial adenocarcinoma, respectively (96, 97). Being derived from cancers, it is also difficult to make direct comparisons between these cell lines and phenotypically “normal” endometrial cells, made more complicated with the continual passage of these cells. This is an advantage of using primary cells, because these cells more accurately reflect *in vivo* conditions. However, many of the primary cells used in the included studies were collected from infertile women with known pathologies, or from women with an unknown or undefined fertility status. While this reflects the difficultly of obtaining normal fertile human endometrial tissue, drawing conclusions from these studies may be unreliable and not directly translatable to endometrial receptivity in normal fertile women. Moreover, drawing accurate comparisons between adhesion assay results across the included studies was difficult due to the use of human or B6C3F1 mice blastocysts, or spheroids made with the JAR, HTR-8/SVneo, or L2TSC cell lines, for the same reasons previously discussed. The JAR, HTR-8/SVneo, and L2TSC cell lines are derived from invasive human choriocarcinoma cells, normal first-trimester placenta, and trophectoderm cells from the 32-cell stage blastocyst, respectively.

### Future Studies

Future studies should validate the differences identified between the PMT and EMT in this review. Future research incorporating human participants is required to more completely characterize the unique endometrial and implantation biology of women. In terms of utilizing newer technologies and methods, the use of three-dimensional cell culture models incorporating primary epithelial and stromal cells from fertile women would increase the translatability of results to the normal endometrium. Emerging studies of cryopreservation and recovery of human endometrium has expanded capacity to use primary epithelial cells for the assessment of epithelial cell polarity and could be employed in future studies (98). To progress the studies toward translation into the clinic, standardized analysis of marker PMT signatures and determinations of the functional significance of the PMT makers using appropriate models will facilitate translation. Information from the cancer field has identified that targeting EMT remains challenging and should be considered when investigating translation, however, endometrial remodeling is a normal physiological process and the endometrial luminal epithelium membrane remodeling does possess all the classical features of EMT described in cancer cells. Therefore direct comparison of EMT between the non-transformed endometrium and cancer should reflect these differences and interpreted accordingly.

## Conclusions

To the best of our knowledge, this was the first systematic review analyzing the current literature discussing the PMT and comparing this to the existing literature on the EMT. Many similarities were found between the process of the PMT and EMT. Most notably, alterations to the actin cytoskeleton, remodeling of adherens junctions, differential integrin expression, and stromal-epithelial communication were similar between these processes. However, there were also some differences between the events of the PMT when compared to the EMT, such as between the alterations to tight junctions and mucin expression. This research will provide new insights into the alterations occurring to the endometrial epithelium during the mid-secretory phase to confer endometrial receptivity. This may provide novel biomarkers or therapeutic targets to manage embryo implantation failure, and therefore help more couples achieve pregnancy.

## Data Availability Statement

The original contributions presented in the study are included in the article/supplementary materials. Further inquiries can be directed to the corresponding author.

## Author Contributions

All authors made substantial contributions to the conception of this review and the critical appraisal of the literature summarized herein. SW wrote the manuscript and it was critically evaluated and revised by WZ and ED. All authors contributed to the article and approved the submitted version.

## Funding

This work was supported by a project grant (APP1120689) and a senior research fellowship (#550905) from the National Health and Medical Research Council (NHMRC) of Australia to ED.

## Conflict of Interest

The authors declare that the research was conducted in the absence of any commercial or financial relationships that could be construed as a potential conflict of interest.
